# Risk of suicide and accidental deaths among elderly patients with cognitive impairment

**DOI:** 10.1186/s13195-019-0488-x

**Published:** 2019-04-11

**Authors:** Ji Hyun An, Kyung Eun Lee, Hong Jin Jeon, Sang Joon Son, Sung Yoon Kim, Jin Pyo Hong

**Affiliations:** 1 Department of Psychiatry, Samsung Medical Center, Sungkyunkwan University School of Medicine, 81 Irwon-ro Gangnam-gu, Seoul, 06351 South Korea; 2 Department of Psychiatry, Ajou University School of Medicine, Suwon, South Korea; 3 Department of Psychiatry, Asan Medical Center, Ulsan University School of Medicine, Seoul, South Korea

**Keywords:** Cognitive impairment, Dementia, Suicide, Accidental death, Elderly

## Abstract

**Background:**

The leading causes of death among the elderly with cognitive impairment are unknown. This study aims to estimate the suicide and accidental death rates on the basis of a clinical case registry of patients diagnosed with cognitive impairment.

**Methods:**

The target sample consisted of 10,169 patients diagnosed with dementia or mild cognitive impairment (MCI), who were evaluated at the Clinical Research Center for Dementia of Korea (CREDOS) from January 2005 to December 2013. Information about whether the patients had died from suicide or in any kind of accident by December 31, 2016, was obtained from the database of the National Statistical Office (NSO). The standardized mortality ratio (SMR) and Cox-regression analysis were performed for evaluating the risk of suicide and accidental death as identified by the ICD-10.

**Results:**

The average of the Clinical Dementia Rating Scale (CDR) score (0.68 vs 0.93) was lower, and the age at the time of study registration (71.42 vs 75.68 years) was younger in the suicidal death group, as compared to the accidental death group. The overall SMR for accidental death in cognitively impaired patients (1.44, 95% CI 1.22–1.71) was significantly higher than the general population. Later onset (1.43, 95% CI 1.20–1.71) and older age (2.21, 95% CI 1.04–4.68) increased the risk of accidental death in cognitively impaired patients. According to the dementia subtypes, the SMR for accidental death was higher in both Alzheimer’s disease (1.72, 95% CI 1.36–2.14) and vascular dementia (2.14, 95% CI 1.27–3.38). Additionally, the SMR for accidental death showed an increasing tendency as the CDR score increased (mild 1.80, 95% CI 1.32–2.42, moderate 1.86, 95% CI 1.07–3.03, severe 3.32, 95% CI 1.08–7.76). Unemployment increased the risks of both suicide (3.71, 95% CI 1.54–8.95) and accidental death (2.09, 95% CI 1.20–3.63).

**Conclusions:**

Among people with cognitive impairment**,** the risk of death by suicide did not increase, whereas that of accidental death increased significantly. Preventive strategies for premature mortality in those with cognitive impairment should be implemented from the early stages and should include careful evaluation of the individual risk factors for each type of death.

## Introduction

Cognitive impairment is prevalent in late life and may impact on mortality of the elderly. The World Health Organization (WHO) estimates that the number of people over the age of 60 will be around 2 billion in 2050, while the number of dementia patients is expected to rise rapidly along with the aging population [1]. Current data suggest that the prevalence of age-adjusted dementia in 65-year-olds is over 5% [2], and their mortality is 3.3–6.0 times higher than that of the general population [3].

As South Korea is now an aged society, the concept of “dying well” becomes more important; thus, the major causes of death among the elderly have been receiving greater attention. Apart from medical comorbidities, suicide and accidental death are also prevalent in the elderly, which suggests that their prevention is a priority in terms of public health.

Suicide is one of the most common causes of death in elderly people, with its prevalence ranging from 18 to 20% [4–6]. Additionally, most mental disorders are known to be associated with increased mortality because of suicide [7]. However, there is a lack of understanding of suicide death among those with cognitive impairment such as dementia or MCI. Death by suicide is known to be infrequent in the advanced stages of cognitive impairment, but some reports have shown a higher risk of suicide during the early stages of cognitive impairment [8, 9] when their insight and the ability to plan suicide are intact. Although there are reports that show depressive symptoms are linked to suicide in patients with cognitive impairment [10–13], little is yet known about the relationship between cognitive impairment itself and suicide.

Accidents are also one of the leading causes of all deaths, but have not been of great interest in terms of their relationship with psychiatric disorders. A recent study reported that all mental disorders, including cognitive impairment, substantially increased the risk of accidental death [14]. However, there is hardly much known about the specific risk factors of accidental death and the relationship between severities of cognitive impairment and accidental death.

The aim of this study was to investigate whether the mortality risks due to suicide and accidental death increase and to investigate the causes of death-related factors among patients with cognitive impairment. We also compared the risks of each cause of death in those with cognitive impairment against in those of the general population, where we hypothesized that the rate of accidental death rather than suicide would be higher among patients with cognitive impairment.

## Methods

### Data sources

The target sample was recruited from the Clinical Research Center for Dementia of Korea (CREDOS) from January 2005 to December 2013. The CREDOS is a clinical research group consisting of board-certified neurologists, psychiatrists, and neuropsychologists, who evaluate the multi-hospital-based longitudinal registry of dementia patients, which is the largest clinical dementia cohort in Korea. All CREDOS patients who visited outpatient clinics in 56 participating hospitals for their cognitive impairment were comprehensively evaluated with standardized neuropsychiatric protocol, as described elsewhere [15].

**Fig. 1:**
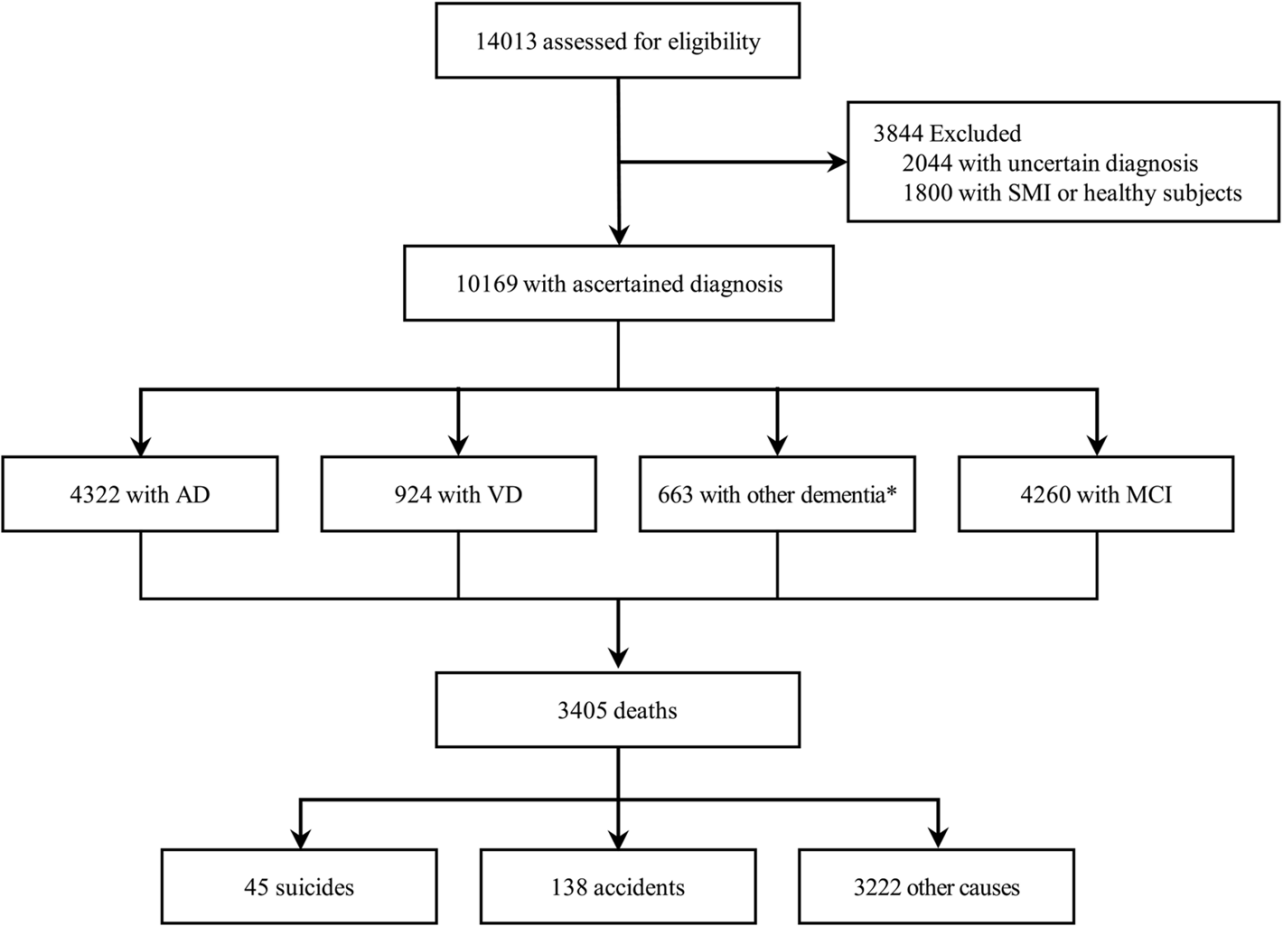
Flow and outcomes of the study patients, 2005 through 2016. AD Alzheimer’s disease, VD vascular dementia, MCI mild cognitive impairment, SMI subject memory impairment. *Other dementia: dementia with Lewy bodies (DLB), frontotemporal dementia (FTD), and unspecified dementia

Exclusion criteria for the CREDOS study were (1) history of significant hearing or visual impairment rendering participation in the interview difficult; (2) neurological disorders (e.g., territorial infarction, intracranial hemorrhage brain tumor, hydrocephalus, multiple sclerosis, Parkinson’s disease, or Huntington’s disease); (3) major psychiatric disorders (e.g., schizophrenia, intellectual disability, current episodes of major depressive disorder, or mania); and (4) physical illnesses that could interfere with the clinical study (e.g., severe cardiorespiratory disease, fulminant hepatic or renal disease, uncontrolled diabetes, or malignancy). This was because such patients had difficulty in receiving the neuropsychiatric interviews or in making periodic visits due to poor medical conditions. A total of 14,013 patients with cognitive impairment who were eligible to enroll in the CREDOS study were selected. Of these, 2044 patients with uncertain diagnosis of cognitive impairment and 1800 healthy subjects with subjective memory impairment were excluded. Finally, a total of 10,169 patients with cognitive impairment were selected for our analysis (Fig. 1). The cohort patients contributed a total of 65,255 person-years, and the mean (SD) and median follow-up time were 6.42 (2.36) and 6.77 years, respectively.

The causes of death of subjects were verified by linking the CREDOS data to the Korean National Statistical Office (NSO) death index database [5] through a unique national identification number assigned to every South Korean citizen since December 31, 2016. Suicide death was indicated by the ICD-10 code as X60–X84 (intentional self-harm), and accidental death was indicated by the ICD-10 codes as W00–W19 (falls), V01–V99 (transport accidents), and X40–X49 (accidental poisoning).

### Measures

#### Diagnosis and classifications of incident cognitive impairment

Patients were diagnosed with cognitive impairment according to the National Institute of Neurological and Communicative Disorders and Stroke, and the AD and Related Disorders Association (NINCDS-ADRDA) [16]; National Institute of Neurological Disorders and Stroke, and Association Internationale pour la Recherche et I’Enseignement en Neurosciences (NINDS-AIREN) [17]; Modified Hachinski Ischaemic Score (MHIS) [18]; McKeith criteria for dementia with Lewy bodies (DLB) [19]; the Lund and Manchester criteria for frontotemporal dementia (FTD) [20]; and the Diagnostic and Statistical Manual of Mental Disorders, Fourth edition (DSM-IV) [21]. For MCI, the criteria in the CREDOS study were as follows: (1) presence of memory-related complaints; (2) intact functioning in activities of daily living (ADL); (3) objective cognitive impairment (at least 1.0 standard deviation [SD] below age- and education-adjusted norms) in more than one cognitive domain on standardized neuropsychological testing [22]; (4) Clinical Dementia Rating (CDR) [23] of 0.5; and (5) considered not demented according to the Diagnostic and Statistical Manual of Mental Disorders (DSM)-IV criteria.

Patient’s sociodemographic variables included sex, age, occupational status, educational level, and duration to death after diagnosis of cognitive impairment and were collected at the time of registration. The ages of patients at the time of registration was designated as their age on the date on which the clinical neuropsychological evaluation was first made for incidents of cognitive impairment. Each participant was entitled to only one major type of cognitive disorder in this study.

#### Clinical evaluation

All study patients underwent a complete medical interview and neuropsychiatric evaluation. Clinical assessments related to the severity of cognitive impairment, including the Clinical Dementia Rating Scale (CDR) and the Korean Mini-Mental State Examination (K-MMSE) [24], were measured by board-certified neuropsychiatrists in face-to-face interviews of each patient. Reliable caregivers of each patient were made to complete several caregiver questionnaires, such as Seoul Instrumental ADL (S-IADL) [25] and the Korean version of the Neuropsychiatric Inventory (K-NPI) [26].

This study was approved by the Institutional Review Board of Samsung Medical Center in Seoul, Korea (IRB No. SMC 2018-08-136). All study patients were fully informed of the aims and methods of the study, and written consent was obtained from all patients.

### Data analysis

The standardized morality ratio (SMR) was computed as the ratio of the observed number of suicides and accidental deaths in those with cognitive impairment to the expected number of each type of deaths, based on the age-, sex-, and time-specific incidence rates among the general Korean population, in the period between January 2005 and December 2016.

The sociodemographic characteristics and findings of the clinical assessments were compared using a Pearson chi-squared test for categorical variables, and Student’s *t* test or Mann–Whitney *U* test for continuous variables. Furthermore, Cox proportional hazards regression analyses were performed to identify the mortality risks of each type of death with the selected variables of cognitive impairment. Patients’ characteristics and clinical variables that were previously demonstrated in univariate analyses to be significant risk factors of each type of death were entered into the multivariate models. All models were initially adjusted for age and sex. We also performed a separate multivariate analysis for the MCI group in order to identify the risk factors of each type of death.

All statistical analyses were performed with the Statistical Analysis System (SAS, version 9.4, SAS Institute Inc., Cary, NC, USA), with a statistical significance cutoff at an alpha level of 0.05.

## Results

### Demographic characteristics associated with suicide and accidental death in elderly patients with cognitive impairment

The demographics and clinical factors of the study patients at registry from January 2005 to December 2013 are summarized according to the type of death in Table 1. For a 12-year-follow-up period, a total of 3405 deaths were observed among 10,169 patients. Among these, 45 patients (0.44%) died by suicide and 138 (1.36%) died due to accidents. The mean duration between the study registration and death was shorter for suicide deaths (2.80 ± 2.28 year) than accidental deaths (3.74 ± 2.45 year), with a statistical significance of *p* = 0.024.

**Table 1:** Demographic and clinical characteristics of study patients at the time of CREDOS registration

Characteristics	Suicide death	Accidental death	Other	*p*	Post hoc
Male sex, *N* (%)	19 (42.2)	73 (52.9)	3383 (33.9)	< 0.000***	Accidental death > others
Age at time of registration, mean (SD), years	71.42 (7.60)	75.68 (7.54)	72.10 (8.41)	< 0.000***	Accidental death > suicide death, others
Occupational status, *N* (%)	11 (24.4)	23 (16.7)	1579 (15.8)	0.575	
Education, mean (SD), years	4.22 (2.50)	5.41 (2.82)	5.14 (2.81)	0.048*	Accidental death > suicide death
CDR score, mean (SD)	0.68 (0.37)	0.93 (0.62)	0.79 (0.54)	0.004**	Accidental death > Suicide death, others
K-MMSE score, mean (SD)	21.29 (5.49)	20.03 (5.23)	21.76 (6.02)	0.009**	Others > accidental death
S-IADL score, mean (SD)	12.29 (10.23)	17.95 (12.07)	13.78 (11.49)	< 0.000***	Accidental death > suicide death, others
K-NPI score, mean (SD)	13.00 (13.90)	15.21 (20.14)	11.88 (16.03)	0.043*	Accidental death > others

Bonferroni correction for multiple comparisons

*Abbreviation: CDR* Clinical Dementia Rating Scale, *CREDOS* Clinical Research Center for Dementia of Korea, *K-MMSE* Korean Mini-Mental State Examination, *SD* standard deviation, *S-IADL* Seoul Instrumental Activity of Daily Living, *K-NPI* Korean version of Neuropsychiatric Inventory

*p < 0.05, **p < 0.01, ***p < 0.001

Around half of the accidental death patients were male (52.9%). Compared to the accidental death group, those in the suicidal death group were younger at the time of study registration and tended to have fewer symptoms of cognitive impairment, which were measured using CDR and S-IADL. The suicidal death group also demonstrated a lesser number of years of education (4.22 vs 5.41 years) and were more frequently employed (24.4 vs 16.7%) than the accidental death group.

### Comparison of SMR for suicide and accidental death in elderly patients with cognitive impairment

Table 2 shows the SMR for suicide and accidental death on the basis of gender, age, and type of impairment at the time of study registration. The overall SMR calculated in patients with cognitive impairment was 0.98 (95% CI 0.71–1.31,) for suicide deaths and 1.44 (95% CI 1.22–1.71) for accidental deaths. Both male (1.71, 95% CI = 1.34–2.15) and female (1.36, 95% CI 1.05–1.73) patients demonstrated higher SMR for accidental deaths. There was no gender difference for suicide deaths. The SMR for accidental deaths was significantly higher when a patient’s age at the time of registration was older (1.43, 95% CI 1.20–1.71).

**Table 2:** Comparison of SMR for suicide and accidental death according to gender, age, and type of cognitive impairment at the time of CREDOS registration

Variables	PYAR	Suicide death			Accidental death		
		Observed death, *N*	Expected death, *N*	SMR (95% CI)	Observed death, *N*	Expected death, *N*	SMR (95% CI)
All (*N* = 10,169)	65,255	45	46.01	0.98 (0.71–1.31)	138	95.61	1.44 (1.22–1.71)
Gender							
Men (*N* = 3475)	20,429	19	24.06	0.79 (0.48–1.23)	73	42.71	1.71 (1.34–2.15)
Women (*N* = 6694)	44,826	26	18.34	1.42 (0.93–2.08)	65	47.76	1.36 (1.05–1.73)
Age at time of registration							
< 65 years (*N* = 1721)	12,211	5	4.96	1.01 (0.33–2.35)	10	6.34	1.58 (0.76–2.90)
65 years ≤ (*N* = 8447)	53,034	40	41.03	0.97 (0.70–1.33)	128	89.26	1.43 (1.20–1.71)
Type of cognitive impairment							
Alzheimer’s dementia (*N* = 4322)	26,291	21	20.23	1.04 (0.64–1.59)	78	45.46	1.72 (1.36–2.14)
Vascular dementia (*N* = 924)	5320	3	3.97	0.75 (0.16–2.21)	18	8.41	2.14 (1.27–3.38)
Other dementia* (*N* = 663)	4070	3	2.43	1.23 (0.67–1.47)	4	4.46	0.90 (0.24–2.30)
Mild cognitive impairment (*N* = 4260)	29,574	18	19.36	0.93 (0.55–1.47)	38	37.28	1.02 (0.72–1.40)

*Abbreviation: CI* confidence interval, *CREDOS* Clinical Research Center for Dementia of Korea, *PYAR* person-years at risk, *SMR* standardized mortality ratio

*Other dementia: dementia with Lewy bodies (DLB), frontotemporal dementia (FTD), and unspecified dementia

Based on the type of cognitive impairment, the SMR for accidental deaths was significantly higher for both Alzheimer’s disease (1.72, 95% CI 1.36–2.14) and vascular dementia (2.14, 95% CI 1.27–3.38).

**Fig. 2:**
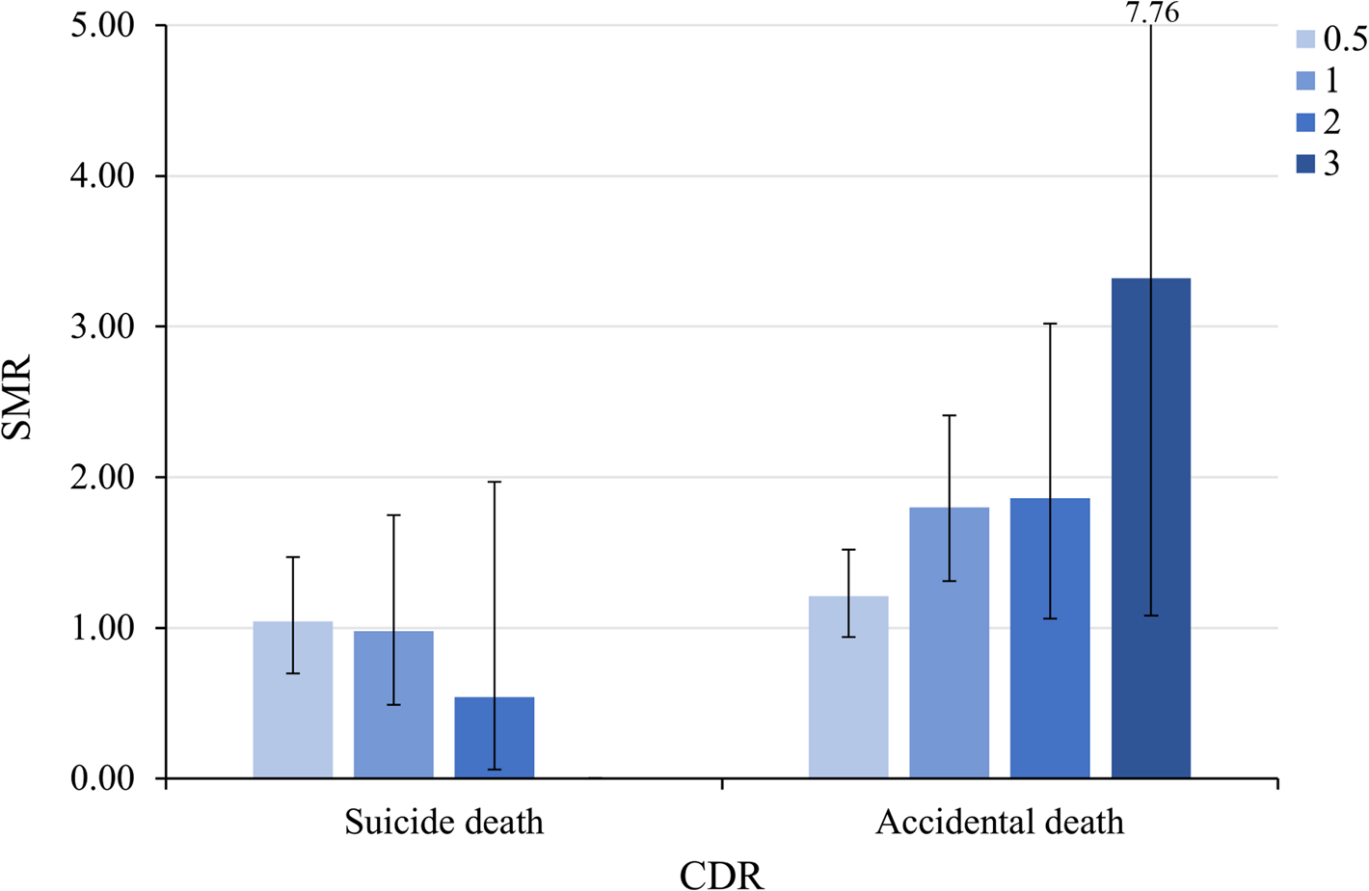
Comparison of SMRs for suicide and accidental death according to the severity of cognitive impairment measured in CDR. CDR Clinical Dementia Rating Scale, SMR standardized mortality ratio

The SMR according to the severity of cognitive impairment, which was measured using CDR, is shown in Fig. 2. The SMR for suicide showed a decreasing tendency as the CDR scores increased (very mild 1.04, 95% CI 0.71–1.48, mild 0.98, 95% CI 0.49–1.75, moderate 0.54, 95% CI 0.07–1.97); however, it was not statistically significant. On the other hand, the SMR for accidental death showed an increasing tendency as the CDR scores increased (mild 1.80, 95% CI 1.32–2.42, moderate 1.86, 95% CI 1.07–3.03, severe 3.32, 95% CI 1.08–7.76).

### Clinical variables associated with suicide and accidental death in elderly patients with cognitive impairment

Table 3 summarizes the results of the Cox regression of the clinical variables associated with suicide and accidental death in dementia and MCI patients.

**Table 3:** Univariate and multivariate Cox proportional hazards models for variables associated with suicide and accidental death in study patients

Variable	HR (95% CI)			
	Suicide death		Accidental death	
	Univariate	Multivariate	Univariate	Multivariate
Total dementia				
Age, years^a^	4.24 (0.58–31.28)	5.14 (0.69–38.50)	2.31 (1.07–4.99)^†^	2.21 (1.04–4.68)^†^
Occupational status				
Yes	Reference	Reference	Reference	Reference
No	2.76 (1.16–6.51)^†^	3.71 (1.54–8.95)^†^	2.06 (1.19–3.59)^†^	2.09 (1.20–3.63)^†^
Education, years^a^	0.96 (0.88–1.03)	0.96 (0.89–1.03)	1.00 (0.97–1.05)	1.01 (0.98–1.05)
Clinical neuropsychiatric scores				
K-MMSE^b^	1.02 (0.84–1.06)	1.01 (0.95–1.09)	1.00 (0.96–1.05)	0.99 (0.96–1.03)
S-IADL^a^	0.98 (0.95–1.03)	0.99 (0.95–1.02)	1.02 (1.01–1.04)^†^	1.06 (1.01–1.08)^†^
K-NPI^a^	1.88 (0.86–4.15)	1.01 (0.99–1.03)	1.01 (1.00–1.02)^†^	1.00 (0.99–1.01)
Mild cognitive impairment				
Age, years^a^	1.07 (0.03–3.27)	1.07 (0.35–3.25)	3.81 (1.02–12.74)^†^	3.63 (1.12–11.79)^†^
Occupational status				
Yes	reference	reference	reference	reference
No	0.99 (0.13–3.21)	0.96 (0.32–2.90)	0.79 (0.33–1.89)	0.76 (0.34–1.73)
Education, years^a^	0.90 (0.82–0.99)^†^	0.90 (0.81–0.99)^†^	1.07 (1.01–1.13)^†^	1.01 (1.00–1.13)^†^
Clinical neuropsychiatric scores				
K-MMSE^b^	1.04 (0.88–1.20)	1.04 (0.91–1.19)	1.03 (1.00–1.15)^†^	1.05 (0.95–1.15)
S-IADL^a^	0.99 (0.89–1.10)	0.97 (0.87–1.07)	1.10 (1.02–1.14)^†^	1.08 (1.03–1.12)^†^
K-NPI^a^	0.94 (0.91–1.05)	0.99 (0.94–1.05)	1.01 (1.00–1.05)^†^	1.02 (1.00–1.05)^†^

*Abbreviation: CI* confidence interval, *HR* Hazard ratio, *K-MMSE* Korean Mini-Mental State Examination, *K-NPI* Korean version of Neuropsychiatric Inventory, *S-IADL* Seoul Instrumental Activity of Daily Living

^†^The hazard ratio was significant (*p* < 0.05)

^a^The hazard ratio is for each 1-year increase in age or 1-point increase in clinical scores

^b^The hazard ratio is for each 1-point decrease in clinical scores

In the dementia group, older age, unemployment, low IADL performance, and more neuropsychiatric symptoms were likely to have increased risk of accidental death in the univariate model. In multivariate analysis, advanced age (HR 2.21, 95% CI 1.04–4.68) and decreased activity of daily living (HR 1.06, 95% CI 1.01–1.08) were associated with a higher risk of accidental death. Unemployment increased the risk of both suicide (HR 3.71, 95% CI 1.54–8.95) and accidental death (HR 2.09, 95% CI 1.20–3.63).

In the MCI group, advanced age (HR 3.63, 95% CI 1.12–11.79), decreased activity of daily living (HR 1.08, 95% CI 1.03–1.12), and more neuropsychiatric symptoms (HR 1.02, 95% CI 1.00–1.05) were associated with a higher risk of accidental death in the multivariate model. Interestingly, as the duration of education increased, the risk of suicide (HR 0.90, 95% CI 0.81–0.99) decreased, while that of accidental death (HR 1.01, 95% CI 1.00–1.13) slightly increased.

## Discussion

To our best knowledge, this is the first cohort study on mortality according to suicide and accidental death that considers the characteristics of each cause of death in patients with cognitive impairment. It is also noteworthy that by separately performing analysis for mortality of the MCI group, we were able to better understand the characteristics of suicide and accidental death in early or milder forms of cognitive impairment. Along with follow-up data for a decade related to causes of death, our findings would contribute toward broadening knowledge of the underlying determinants of suicide and accidental death in patients with cognitive impairment.

According to our study, the risk of suicide among patients with cognitive impairment did not increase significantly, which is consistent with the findings of most previous studies [27–29]. Even though there have been several efforts to predict the clinical factors of suicidal death in people with dementia, most participants were either psychiatric inpatients or had severe medical comorbidity [13, 30, 31], which made it difficult to ascertain the true risks of suicide itself in those with cognitive impairment. Furthermore, suicide in elderly patients with cognitive impairment did not demonstrate the usual risk factors for elderly suicide, such as the male gender or advancing age [32]. This suggests that the nature of suicide in elderly patients with cognitive impairment is somewhat different from the elderly without cognitive impairment.

However, among the suicide cases, cognitive limitation seems to be a hindrance to the implementation of a suicidal plan. Our study shows that the suicidal death group was younger and had better cognitive function, and their mean time from registry to death was shorter. This leads us to believe that they may have had less cognitive impairment and more preserved insight at the time death. This supports the findings of several previous studies [8, 33] that report better cognitive function enabling suicidal execution, and retained insight into the disease may increase the risk of dying by suicide in dementia patients [11]. As an interesting comparison, cancer patients were more likely to die from non-cancer causes than from cancer itself [34]. Recent studies consistently reported that the risk of suicide among cancer patients is higher than that of the general population and younger cancer patients within the first year of diagnosis had higher suicide death [35, 36]. Both dementia and cancer patients with better insight may possibly become frustrated due to their awareness of functional impairment or loss of autonomy. Therefore, close observation and suicide prevention strategies have to be aimed at such patients.

On the other hand, the risk of accidental death tends to increase as CDR and the severity of cognitive impairment increase in both dementia and MCI groups, which might be due to more impaired locomotor activity, such as reaction time, coordination, or walking as well as deterioration of situational awareness and short-term memory. This finding suggests that the cause of death may vary according to the severity of neurocognitive impairment in patients with cognitive impairment.

Older age was a consistent risk factor for accidental death in both dementia and MCI groups. It is noteworthy that the mortality rate of accidental death is more than 3.5 folds in MCI by 1 year. Aging seems to be an independent risk factor of accidental death in people with a cognitive impairment; therefore, we should be aware of the potential risk of accidents beginning from the early stages of the disease. Interestingly, current unemployment state increased the risk of both suicide and accidental death in dementia patients. Leaving employment is reported to be a risk factor of suicide [37]. Furthermore, patients with advanced cognitive impairment symptoms may be unable to continue working; hence, unemployment can be one of the predictors of decreased functional ability, which may subsequently increase the risk of accidents. According to the subtypes of cognitive impairment, both Alzheimer’s disease and vascular dementia demonstrated high SMR for accidental death. Patients with vascular dementia had over two folds of the risk of accidental death, which could be due to cardiovascular risk or medical comorbidity of vascular dementia itself.

As we had expected, suicide and accidental deaths are the primary issues that need to be considered to reduce premature mortality in elderly patients with cognitive impairment and are related to several clinical risk factors.

There are a few limitations of this study. First, as we included clinical variables at the time of study registration, it enabled clinicians to screen mortality risk among patients with cognitive impairment initially, but there was a lack of timely details of death-related events. For example, a patient may have had depression or medical comorbidities that had an independent effect on his or her mortality at the time of death. Second, the small number of suicidal deaths limited the study of risk factors of death. Further research should focus on elucidating the temporal relationship between each type of death and dementia-specific symptoms, in addition to suicidal ideation or attempts, self-mutilating behaviors, and non-fatal accidents.

## Conclusions

The risk of death by suicide did not increase significantly among patients with cognitive impairment. However, the risk of accidental death was higher, especially in older patients and those with severe forms of cognitive impairment. Improved awareness and understanding of patients’ mortality are needed at the onset of cognitive evaluation in order to develop better risk-management strategies.

## Abbreviations

CDR: Clinical Dementia Rating Scale
CREDOS: Clinical Research Center for Dementia of Korea
K*-*MMSE: Korean Mini-Mental State Examination
MCI: Mild cognitive impairment
NPI: Neuropsychiatric inventory
S-IADL: Seoul Instrumental Activity of Daily Living
SMR: Standardized morality ratio
